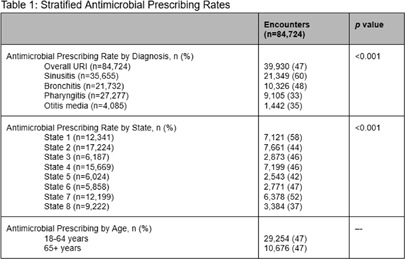# Regional Variation Impacts Outpatient Antimicrobial Prescribing for Adults with Upper Respiratory Infections in a Large Health System

**DOI:** 10.1017/ash.2025.273

**Published:** 2025-09-24

**Authors:** Reese A. Cosimi, Melinda Mackey, Maria Mupanomunda, Julie Hunt, Ashlin Jones, Aaron Shoemaker, Stacy Garrett-Ray, Frederick A. Masoudi, Thomas Aloia, Mohamad Fakih

**Affiliations:** 1 Ascension, St. Louis, MO; 2 Ascension Data Sciences Institute, Ascension, St. Louis, MO; 3 University of Maryland School of Medicine, Baltimore, MD; 4 Wayne State University School of Medicine, Detroit, MI

## Abstract

**Background:** Upper respiratory infections (URIs) are a common cause of outpatient visits in adults. While most URIs are viral, antimicrobial prescribing rates remain high. The COVID-19 pandemic disrupted usual practices, necessitating an evaluation of the post-pandemic landscape for antimicrobial prescribing for URIs. This study sought to characterize factors contributing to variability in utilization in a large multi-state health system. **Methods:** Retrospective analysis of antimicrobial prescribing in patients ≥18 years of age for URI diagnosis codes in 863 outpatient sites (eight states), including office visits, urgent care, and telemedicine between July 1, 2023 to June 30, 2024. Primary outcome was antimicrobial prescribing rates for URIs overall and by individual URI diagnosis (sinusitis, bronchitis, pharyngitis, otitis media). HEDIS definitions were applied where appropriate. Logistic regression machine learning models were used with SHapley Additive exPlanations (SHAP) analysis to show feature contributions to antimicrobial prescribing. **Results:** A total of 84,724 patient encounters were included with four URI diagnoses. Antimicrobial prescribing rates varied by diagnosis (sinusitis: 60%, bronchitis: 48%, pharyngitis: 33%, otitis media: 35%, p<0.001). Prescribing ranged from 37%-58% across states (p<0.001). Sinusitis diagnosis and specific states had the strongest positive associations with antimicrobial prescribing, while race and social vulnerability index (SVI) were not associated. **Conclusions:** In this study in a large multi-state US health system, antimicrobials were most commonly prescribed for patients with sinusitis. Regional variation was also associated with increased prescribing. These data support efforts to standardize practices and address clinical variation.

**Figure:**
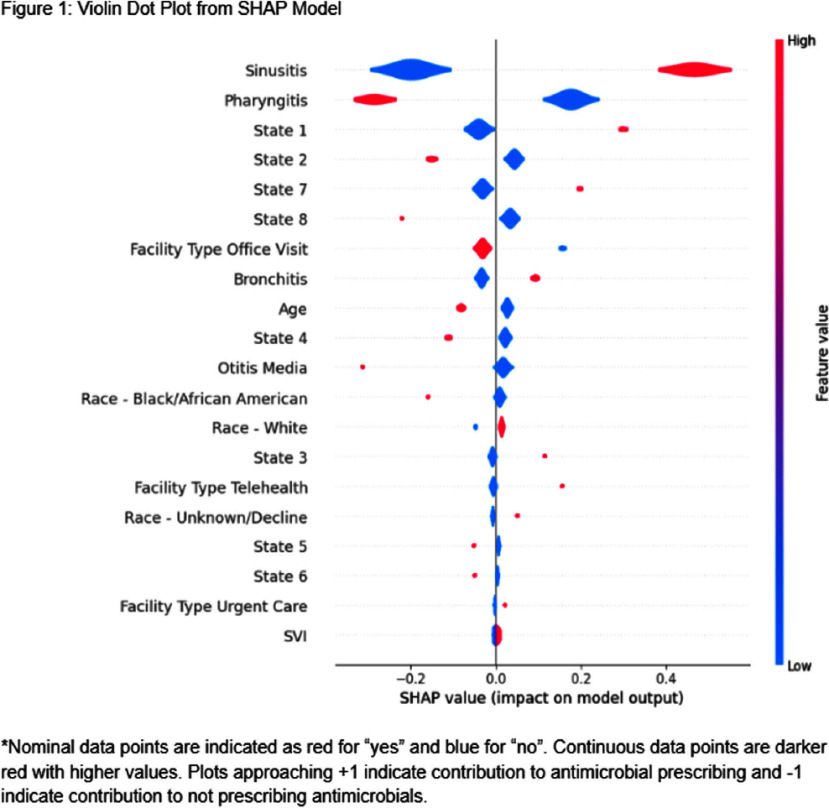

**Figure:**